# The brain-derived neurotrophic factor Val66Met polymorphism is associated with reduced functional magnetic resonance imaging activity in the hippocampus and increased use of caudate nucleus-dependent strategies in a human virtual navigation task

**DOI:** 10.1111/j.1460-9568.2010.07550.x

**Published:** 2011-03

**Authors:** Harrison Banner, Venkataramana Bhat, Nicole Etchamendy, Ridha Joober, Véronique D Bohbot

**Affiliations:** Department of Psychiatry, Faculty of Medicine, McGill University, Douglas Mental Health University InstituteFBC Building, 6875 boul. LaSalle, Verdun, QC H4H 1R3, Canada

**Keywords:** BDNF polymorphism, caudate nucleus, fMRI, hippocampus, navigational strategy, spatial memory

## Abstract

Multiple memory systems are involved in parallel processing of spatial information during navigation. A series of studies have distinguished between hippocampus-dependent ‘spatial’ navigation, which relies on knowledge of the relationship between landmarks in one’s environment to build a cognitive map, and habit-based ‘response’ learning, which requires the memorization of a series of actions and is mediated by the caudate nucleus. Studies have demonstrated that people spontaneously use one of these two alternative navigational strategies with almost equal frequency to solve a given navigation task, and that strategy correlates with functional magnetic resonance imaging (fMRI) activity and grey matter density. Although there is evidence for experience modulating grey matter in the hippocampus, genetic contributions may also play an important role in the hippocampus and caudate nucleus. Recently, the Val66Met polymorphism of the brain-derived neurotrophic factor (BDNF) gene has emerged as a possible inhibitor of hippocampal function. We have investigated the role of the BDNF Val66Met polymorphism on virtual navigation behaviour and brain activation during an fMRI navigation task. Our results demonstrate a genetic contribution to spontaneous strategies, where ‘Met’ carriers use a response strategy more frequently than individuals homozygous for the ‘Val’ allele. Additionally, we found increased hippocampal activation in the Val group relative to the Met group during performance of a virtual navigation task. Our results support the idea that the BDNF gene with the Val66Met polymorphism is a novel candidate gene involved in determining spontaneous strategies during navigation behaviour.

## Introduction

The existence of multiple memory systems has been demonstrated in both animals and humans (Scoville & Milner, 1957; O’Keefe & Nadel, 1978; Packard *et al.*, 1989; Squire & Zola-Morgan, 1991; McDonald & White, 1993, 1994; Alvarez *et al.*, 1995; Packard & McGaugh, 1996; Wolbers *et al.*, 2004; Milner, 2005; Wolbers & Buchel, 2005), with the hippocampus playing a role in episodic, relational and spatial memory, and the caudate nucleus being implicated in procedural learning and the automatization of behaviour or habit formation. Most evidence suggests that these two systems function largely independently of one another (Bohbot *et al.*, 2004; Mizumori *et al.*, 2004).

Navigating in one’s environment can be achieved by using either of these two memory systems, each of which is responsible for a different strategy (Packard *et al.*, 1989; McDonald & White, 1993, 1994; Packard & McGaugh, 1996; Maguire *et al.*, 1998; Hartley *et al.*, 2003; Iaria *et al.*, 2003; Bohbot *et al.*, 2004; Mizumori *et al.*, 2004; Voermans *et al.*, 2004; Hartley & Burgess, 2005). A ‘spatial’ strategy involves building relationships between landmarks in the environment in order to develop a cognitive map, and is associated with increased grey matter and activity in the hippocampus (Iaria *et al.*, 2003; Bohbot *et al.*, 2004), whereas a ‘response’ strategy involves the learning of stimulus–response relationships, such as a series of turns from specific points in space. The response strategy is associated with increased grey matter and significant brain activity in the caudate nucleus (Bohbot *et al.*, 2007).

Recent work provides evidence that a valine (Val) to methionine (Met) single nucleotide substitution at codon 66 of the brain-derived neurotrophic factor (BDNF) gene is associated with decreased secretion of the BDNF protein (Egan *et al.*, 2003; Bath & Lee, 2006), a polypeptide growth factor involved in neuronal cell survival and differentiation during development. BDNF is implicated in the regulation of synaptic plasticity, and a critical amount is necessary for long-term potentiation in hippocampal CA1 synapses (Figurov *et al.*, 1996). A decrease in BDNF level may lead to impairment of hippocampus-dependent behaviours, such as episodic and spatial recognition memory (Egan *et al.*, 2003; Hariri *et al.*, 2003; Dempster *et al.*, 2005). Individuals with one or two copies of the Met allele exhibited decreased hippocampal functional magnetic resonance imaging (fMRI) function (Hariri *et al.*, 2003) and grey matter (Pezawas *et al.*, 2004; Szeszko *et al.*, 2005; Bueller *et al.*, 2006) as compared with homozygous Val individuals.

Because of BDNF’s role in normal hippocampal function, we predicted that a single nucleotide polymorphism in the BDNF gene would contribute to individuals preferentially using a caudate nucleus-dependent response strategy at the expense of a hippocampus-dependent spatial strategy in the four-on-eight virtual maze (4/8VM), a virtual navigation paradigm designed to differentiate between the two strategies (Bohbot *et al.*, 2004, 2007). We also predicted that individuals with one or two copies of the Met allele would show reduced hippocampal and increased caudate nucleus fMRI activity relative to homozygous Val participants on the fMRI concurrent spatial discrimination learning task (CSDLT), owing to the spontaneous use of response strategies.

## Materials and methods

This study was divided into two components. One hundred and six volunteer participants (53 women and 53 men) aged 18–35 years, with a mean education duration of 15.8 years (standard deviation, ±2.09 years), were recruited to the Douglas Mental Health University Institute at McGill University [Val group: mean age, 23.4 years; standard error of the mean (SEM), 1.1 years; 32 men and 35 women] [Met group: mean age, 22.7 years; standard error of the mean (SEM), 2.3 years; 21 men and 18 women] and completed the behavioural component. Of these, 21 also participated in the fMRI component of the study. All potential participants were given a phone screening questionnaire to assess inclusion/exclusion criteria. Participants had no personal or immediate family history of primary degenerative neurological disorders, and no personal history of neurological or psychiatric diagnosis, alcohol abuse or drug abuse. No participants were taking any medication or had known conditions that could affect cerebral blood flow (e.g. anti-cholesterol medication). The study was approved by the institutional review boards at McGill University, the Douglas Mental Health University Institute, and the Montreal Neurological Institute. All participants who took part in the brain imaging components of the study were right-handed, had normal or corrected-to-normal vision, and were screened for claustrophobia and metallic implements in their body. Informed consent was obtained from each participant prior to commencement of behavioural testing and again before brain imaging.

Blood samples were collected at the Douglas Mental Health University Institute, and BDNF Val66Met genotyping was performed in the laboratory of R. Joober and at Génome Québec. The genotyping was performed after the completion of the behavioural and imaging studies, so both participants and experimenters were blind to the genotype status. Polymerase chain reaction amplification was performed in the laboratory of R. Joober, with the ABI PRISM SNaPshot Multiplex Kit assay (Applied Biosystems, Foster City, CA, USA). BDNF Val66Met genotyping was then performed at Genome Quebec, with TaqMan chemistry (Applied Biosystems). A fluorogenic probe used in TaqMan chemistry enables the detection of a specific polymerase chain reaction product, and differentially labelled fluorogenic probes allow allelic discrimination. The fluorescence emission detection is performed with the 7900HT reader, and the TaqMan technology has an average conversion rate of approximately 98%, with an average error rate of 0.1%. All of the genotypes were in line with Hardy–Weinberg equilibrium, and the BDNF Val66Met genotype distribution was comparable to that reported in other population-based cohorts. All the subjects were genotyped (*n* = 106), and none was excluded because of missing genotype values. Furthermore, there were no demographic differences (age and gender) between the genotypes.

### Experiment 1: behavioural study

One hundred and six participants were tested on the 4/8VM, and completed a battery of neuropsychological tests, which included the Shipley Institute of Living Scale, the Rey Auditory Verbal Learning Task (RAVLT), and the Rey–Osterrieth Complex Figure Task, to assess cognitive function.

The Shipley Institute of Living Scale has been used previously to assess general intellectual functioning and to aid in the detection of cognitive impairment (Zachary, 2000). The RAVLT consists of word list learning, spontaneous retrieval, and recognition-type retrieval with the aid of written word lists. This task provides an assessment of immediate memory, efficiency of learning, effects of interference, and recall following short and long delay periods (30 s and 30 min) (Lezak, 1995). The Rey–Osterrieth Complex Figure Test is widely used for the assessment of visuospatial processing, memory, and executive function (Lezak, 1995). These tasks were used to assess cognitive performance. None of the participants performed at below-normal (cognitively impaired) levels, as defined by Lezak (1995) and Zachary (2000), on any of these tasks, and thus no participants were excluded from further testing on this basis.

**Fig. 1:**
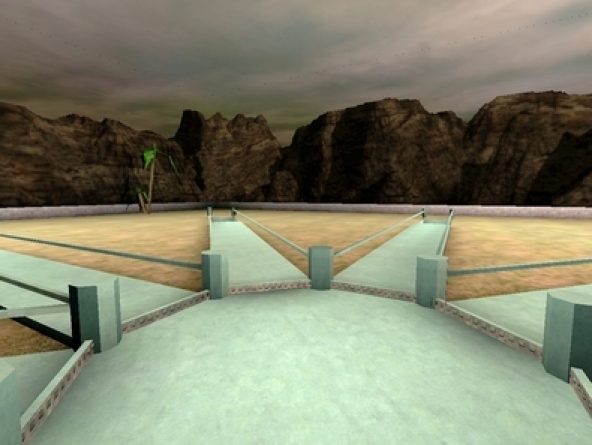
Visual depiction of the 4/8VM environment.

The 4/8VM is a virtual reality navigation task that is performed on a computer, and consists of the participant walking through an enriched and controlled virtual environment. This task was created by using software from a commercially available computer game (Unreal; Epic Games, Raleigh, NC, USA) and has been described in previous studies (Iaria *et al.*, 2003; Bohbot *et al.*, 2004, 2007). A visual representation of the virtual environment is presented in Fig. 1. Briefly, the virtual environment consists of an eight-arm radial maze with a central starting location. The maze is surrounded by a landscape and distal landmarks, namely two trees, a sunset, and mountains. At the end of each of the eight arms is a set of stairs leading down to a pit, where an object can be picked up. Prior to testing, participants are trained in a different virtual environment on the use of a keypad, which allows them to move around during the task.

Participants perform five trials, each of which consists of two separate parts. In Part 1, four of the eight arms are accessible, with objects at the end of each arm. The other arms are blocked by a barrier. In Part 2, all arms are accessible, and objects are now present in the four arms that were blocked in Part 1. Prior to starting each trial, participants are instructed to retrieve all four objects from the accessible arms in Part 1 and to remember which arms have been visited, so that they can avoid these arms in Part 2. In the first three trials, landmarks are visible in both Parts 1 and 2. Participants are able to solve the task by using either a ‘spatial’ strategy, which is dependent on the relationships between objects in the environment, for example by remembering the position of target objects in relation to the two trees and the sunset, or a ‘response’ strategy, in which the series of arms to be visited is memorized with the use of a pattern or numbering system, for example ‘go straight ahead from the start position and take the first left upon exiting the arm’. In Part 1 of the fourth trial, all landmarks are visible, and again there are four blocked arms; however, in Part 2, a wall is erected around the radial maze, blocking the participant’s view of the distal environment. This makes it difficult for participants who use a spatial strategy, as they can no longer use the landmarks to locate the objects in the maze. On the other hand, participants who use a response strategy are not affected by the removal of landmarks, because they were not using them in the first place. Thus, the fourth trial serves as a probe trial to differentiate between participants using a spatial strategy and those using a response strategy; the rationale for the probe being that if participants were using a spatial strategy, this change in the environment should result in an increase in errors, whereas the use of a response strategy would not (Iaria *et al.*, 2003). In the fifth trial, landmarks are again visible in both Parts 1 and 2. This final trial after the probe allows us to see whether participants who used a spatial strategy in the previous trials will switch to a response strategy after the probe trial.

At the end of the task, the participant is debriefed and asked to report how they solved it. As in previous studies (Iaria *et al.*, 2003; Bohbot *et al.*, 2004, 2007), participants were categorized as ‘response’ learners when they reported associating the arms with numbers or letters or counting the arms from a single starting point. Participants were considered to be ‘spatial’ learners if they mentioned using at least two landmarks and did not mention counting and labelling arms. For the purposes of this study, we assessed spontaneous strategies by classifying participants according to their ‘initial strategy’. Initial strategy was defined as the strategy spontaneously used at the beginning of the task in the event that the participant reported having started with one strategy and then shifted to the other.

Dependent variables included the latencies for each trial of the 4/8VM (excluding probe trials, which are used for strategy identification), the total number of errors made during non-probe 4/8VM trials, Shipley Vocabulary Score, Shipley estimated WAIS-R IQ, RAVLT delayed recall, RAVLT total recall, RAVLT recognition score, and Rey–Osterrieth Delayed Recall score. spss for Windows (version 11.01) was used to conduct a one-way anova and chi-squared analysis on these variables, with navigational strategy and genotype group (Val or Met) as main effects. In order to investigate whether there was a relationship between real-life navigation and spontaneous strategies, driving history data were collected for a subsample of the participants (*N* = 75). We asked participants whether they had driving experience, whether they were comfortable in finding their way around the city, and whether they got lost. For all behavioural analyses, the *a priori* threshold of significance was set at *P* = 0.05.

For the purposes of the behavioural analyses, participants were divided into three distinct genotype groups on the basis of the number of Met alleles (0, 1 or 2). The ‘0’ group had genotype Val/Val, the ‘1’ group had genotype Val/Met, and the ‘2’ group had genotype Met/Met. This method of dividing participants has been used in previous behavioural studies with large sample sizes (Egan *et al.*, 2003), as there is some evidence that the Val66Met polymorphism has a dose-dependent effect on hippocampus-related processes (Hashimoto *et al.*, 2008). The experimental groups did not significantly differ with regard to age or sex.

### Experiment 2: fMRI study

**Fig. 2:**
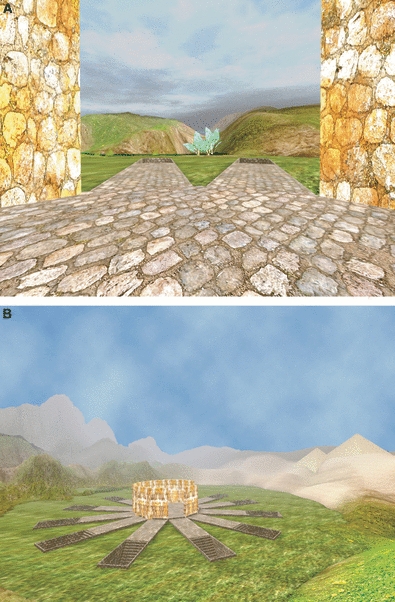
Visual depiction of the CSDLT environment. (A) Participants’ view of the environment, showing one of six pairs of pathways. (B) Overhead view of the environment, showing an enriched distal landscape.

A subsample of 21 participants was tested on the CSDLT while undergoing an fMRI scan. The CSDLT is a virtual navigation task designed with the use of software from a commercially available computer game (Unreal Tournament 2003; Epic Games), and has previously been shown to differentially activate the hippocampus and caudate nucleus in participants using spatial and response strategies, respectively (Etchamendy *et al.*, 2007). A pictorial representation of the virtual environment is shown in Fig. 2. In this task, participants navigate through a virtual environment consisting of a 12-arm radial maze with a central starting location. The maze is surrounded by an enriched landscape with landmarks. At the end of each arm, there is a staircase leading to a pit where, in some of the arms, an object is located. The 12 arms of the maze are divided into six pairs of adjacent arms, with each pair of arms being differentiable on the basis of its proximity to a visible landmark. Within each pair of arms, one arm contains an object and the other arm is always empty. The CSDLT consists of two separate parts, an encoding phase and a test phase. During the encoding phase (Stage 1), the six pairs of arms are repeatedly presented in turn to the participant according to a pseudo-random sequence, and the participant is explicitly asked to learn progressively, within each pair, which arm contains an object and which does not. In the encoding phase, a trial is defined as the presentation of all six pairs of arms. Choice accuracy is measured by the percentage of correct arms chosen. Training continues until the participant reaches a criterion of choice accuracy of at least 91% across two consecutive trials (11 correct arms selected out of 12), with a minimum of six trials being performed. This minimum number of trials ensures that each participant has had the opportunity to properly learn the location of the objects before moving on to the test phase.

**Fig. 3:**
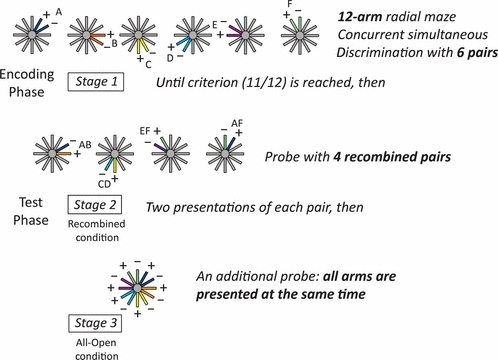
Schematic representation of the CSDLT. Encoding phase: six pairs of pathways are presented individually and repeatedly in a pseudo-random order until participants learn to a criterion of 11/12 correct choices. Test phase recombined condition: reward contingency remains the same, but pairs are recombined into new combinations. Participants complete two trials of four recombined pairs each. Test phase all-open condition: pathways are no longer divided into pairs, and participants have access to the entire environment. Participants are told to collect the objects from the six rewarded pathways while avoiding empty pathways. Each of the letters A, B, C, D, E and F represent a pair of arms presented simultaneously in Stage 1. The letter combinations AB, CD, EF and AF represent the newly recombined pairs of arms in Stage 2. For example, AB represents the combination of one arm from pair A and one arm from pair B.

Following the encoding phase, the participant completes a test phase. The first part of the test phase (Stage 2) consists of the ‘recombined pairs’ condition. During the recombined pairs condition, the reward contingency among the arms remains the same but their presentation is modified, such that the arms are rearranged into novel pairs, and these pairs are presented in a pseudo-random sequence (Fig. 3). Success in this phase requires that the participant previously acquired the task with a spatial strategy by having learned the spatial relationships between the rewarded arms and environmental landmarks as opposed to learning the stimulus-response relationships (e.g. take the left arm when I see the tower). In this way, the test phase acts as a probe for spontaneous navigational strategy. Each participant completes two trials in which only four of the recombined pairs are presented because both of the adjacent arms of the remaining two recombined pairs were empty or contained an object.

The second part of the test phase (Stage 3) is the ‘all-open’ condition. Here, arms are no longer presented as pairs. Instead, all pathways are simultaneously visible and accessible, and the participant is asked to visit all six pathways containing objects while avoiding those that are empty. A schematic diagram showing the different phases of the CSDLT is shown in Fig. 3.

A visuo-motor control task takes place in a different environment from the experimental task but includes a similar radial maze. For this task, the position of the rewarded arms is completely randomized and changed from one trial to another. The participant is presented with pairs of pathways, and is asked to randomly visit arms in order to pick up objects. The experimenter specifies that the locations of the objects are totally randomized and vary across trials, that no rule predicts their positions, and that the participant has nothing to learn. At the same time, the participant is asked to count backwards by increments of 3 from 1000, to discourage rehearsal of information learned during the experimental task. A control equivalent to the all-open condition (Stage 3), in which the participant is asked to visit random arms in a radial maze in which all pathways are available, is presented during the same scan as the experimental all-open condition. In this control condition, the objects are placed at random locations, and thus there is no memory component involved.

The scanning session consisted of several scans with a duration of 10 min each. The number of scans recorded varied across participants, as it was a function of the number of trials needed to attain the criterion performance in the encoding phase. In each scan, participants performed alternate blocks of experimental and visuo-motor control tasks. This was repeated until participants reached the predetermined criterion performance. The testing phase was also interleaved with sequences of the visuo-motor control task. Because of the variability between participants in the time taken to perform the task, custom software was used to record frame times and keystrokes made by the participant and the experimenter (Iaria *et al.*, 2003). Recording the keystrokes of the experimenter marked the transition from one task to another, and allowed us to exclude frames acquired during the transitions between tasks from the analysis.

Magnetic resonance imaging scans were obtained at the Montreal Neurological Institute with a 1.5-T Siemens Sonata scanner. A vacuum cushion was used to stabilize the participant’s head, and the task was presented on a projector outside of the scanner, viewable to the participant through a mirror attached to the head coil. The fMRI scanning session followed a structural scan. It consisted of a sagittal localizer followed by a series of test blood oxygen level-dependent (BOLD) scans. Functional images were acquired with a single-shot T2*-weighted gradient echo EPI pulse sequence (TR, 3000 ms; TE, 50 ms; FOV, 256 mm^2^; matrix size, 64 × 64; in-plane resolution, 2 × 2 mm; 300 whole brain acquisitions per run). Each whole brain acquisition consisted of 32 oblique slices of thickness 4 mm, with a 0.5-mm slice gap, positioned parallel to the hippocampus and covering the entire brain. Structural scans were co-registered with the fMRI scans. Each participant’s fMRI session was divided into three to eight scans of no more than 10 min each, with each scan consisting of both experimental and control trials.

fmristat was used for the statistical analysis of fMRI data (Worsley *et al.*, 2002). BOLD signal images were spatially smoothed with a 6-mm FWHM Gaussian kernel, corrected for motion and linearly transformed into stereotaxic space (Talairach & Tournoux, 1988), using in-house software (Collins *et al.*, 1994). The output of the analysis was displayed as a statistical map, overlaid on an image of a structural magnetic resonance imaging scan. The resulting *t*-statistic images were thresholded by using the minimum given by a Bonferroni correction for multiple comparisons and Gaussian random field theory (Worsley *et al.*, 2002). Restricted search (region of interest) analysis with an uncorrected *P*-value of 0.001 (*N* = 21, *t* = 3.55; *N* = 16, *t* = 3.71; *N* = 5, *t* = 7.17) was used for voxels in the hippocampus and caudate nucleus, because of our strong *a priori* hypothesis that we would observe significant activations in these brain areas. A Bonferroni correction for multiple comparisons was used for all brain areas outside of the hippocampus and caudate nucleus, based on 1 000 000 voxel comparisons (*t* = 6.92).

For the purposes of the fMRI analyses, participants were divided into two distinct genotype groups. Owing to the sample size in the imaging components of the study and the low frequency of Met/Met individuals in the general population (i.e. < 5%; Egan *et al.*, 2003), participants were grouped into either the Val group, consisting of individuals homozygous for the Val allele of the Val66Met polymorphism, or the Met group, which was composed of both homozygous Met individuals and heterozygous individuals who have both a Val and a Met allele. This method of dividing participants has been used in previous imaging studies involving the BDNF Val66Met polymorphism (Hariri *et al.*, 2003; Pezawas *et al.*, 2004; Dempster *et al.*, 2005). These groups did not differ with regard to age or sex.

Experimental trials were contrasted against control trials of equal durations. fMRI scans of the Val and Met groups were contrasted separately. An interaction analysis was then performed, contrasting total activity for the Val group with that of the Met group for each of our selected contrasts of interest. fMRI contrasts from the early and late phase were selected because of our hypotheses that the hippocampus is a fast learning system and is engaged early on (Packard & McGaugh, 1996; Iaria *et al.*, 2003), as opposed to the caudate nucleus, which is a slow learning system that becomes engaged with repetition and habit formation. fmristat was used for correlations between the fMRI signal and other variables. The fMRI signal was covaried with errors and latencies for performance of the task.

## Results

The Val66Met allele frequencies were 0.77 for the Val allele and 0.23 for the Met allele in the behavioural sample, with 36.8% of participants carrying the Met allele (Val/Val, *N* = 67; Val/Met, *N* = 29; and Met/Met, *N* = 10). For the fMRI sample, the Val allele frequency was 0.88 and the Met allele frequency was 0.12, with 23.8% of participants carrying the Met allele (Val/Val, *N* = 16; Val/Met, *N* = 5; Met/Met, *N* = 0). These numbers are consistent with the expected range for North American populations based on previous studies (Shimizu *et al.*, 2004).

### Experiment 1: behavioural study

**Fig. 4:**
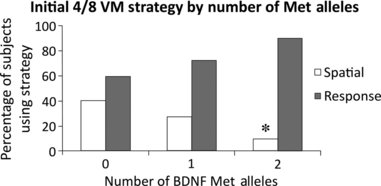
Comparison of initial strategy use on the 4/8VM grouped by number of Met alleles (0 = Val/Val; 1 = Val/Met; 2 = Met/Met). The division is shown by the percentage of participants within a given genotype group. (*) A significant association was observed between the number of Met alleles and initial 4/8VM spontaneous strategy (linear-by-linear association, *χ*^2^ = 4.203, *P* < 0.04), with the proportion of participants using a non-hippocampal response strategy increasing with the number of Met alleles.

Division of participants into groups on the basis of the number of Met alleles (0 = Val/Val; 1 = Val/Met; 2 = Met/Met) and performance of a linear-by-linear association chi-squared analysis of genotype vs. initial strategy revealed a significant relationship between genotype and spontaneous strategies (linear-by-linear association, *χ*^2^ = 4.203, *P* < 0.05) (Fig. 4). *Post hoc* comparisons revealed that there was a significantly higher frequency of subjects with response learning strategies in the Met/Met group than in the Val/Val group (*χ*^2^ = 3.45, *P* < 0.05), which showed a higher frequency of subjects with spatial learning strategies. There was no significant difference between the Met/Met and Met/Val groups (*χ*^2^ = 1.296, *P* = 0.13), or the Met/Val and Val/Val groups (*χ*^2^ = 1.41, *P* = 0.118). Our data confirm that the proportion of participants using response learning strategies increases with the number of Met alleles at codon 66 of the BDNF gene.

As expected, no relationship was observed between Val66Met genotype and other measures of cognition, that is, errors on the RAVLT, Rey–Osterrieth Complex Figure score, or Shipley Institute of Living Scale, or errors on the 4/8VM, using an analysis of variance test with both genotype group (Val or Met) and number of Met alleles (0, 1, or 2) as grouping variables at a significance level of *P* < 0.05. In other words, participants in the Val genotype group performed similarly to participants in the groups with one or two Met alleles on the behavioural test battery. No relationship was observed between subject age, sex or level of education and spontaneous strategies on the 4/8VM or performance on any of the above measures. None of the questions pertaining to driving history, wayfinding and getting lost led to significant chi-square differences between spatial and response learners (driving experience, *χ*^2^ = 0.17, *P* = 0.68; wayfinding, *χ*^2^ = 0.884, *P* = 0.356; getting lost, *χ*^2^ = 0.38, *P* = 0.537). Overall, this is consistent with our previous findings showing that response learners can perform as well as spatial learners on a wayfinding task in a virtual town (Etchamendy & Bohbot, 2007).

### Experiment 2: fMRI study

Participants were divided into two groups on the basis of whether they were homozygous Val carriers or Met carriers. Performance of a Fisher’s exact test analysis of genotype vs. initial strategy revealed no significant differences between the number of spatial and response learners in each genotype group (Val group, spatial = 56.3%, response = 43.8%; Met group, spatial = 80%, response = 20%; *P* = 0.606, Fisher’s exact test).

On the basis of our *a priori* hypotheses regarding the genetic contribution to activity of the hippocampus and caudate nucleus in this task, the restricted search threshold was used. For all voxels located outside of these structures, a Bonferroni correction for multiple comparisons was used, resulting in a higher threshold. No voxel outside of the hippocampus and caudate nucleus crossed this threshold for any of our measured contrasts, and therefore voxels outside the hippocampus and caudate nucleus will not be discussed further.

Consistent with our hypothesis, we found differences in memory system recruitment between Met carriers and the homozygous Val group across both the encoding and test phases of the fMRI CSDLT. Breaking the patterns of brain activation down into early learning, late learning and test phase activity revealed significant activations in the hippocampus early in learning in the Val group but not in the Met group, whereas activation of the caudate nucleus was observed in the Met group but not in the Val group later in the encoding phase and during the test phase.

**Fig. 5:**
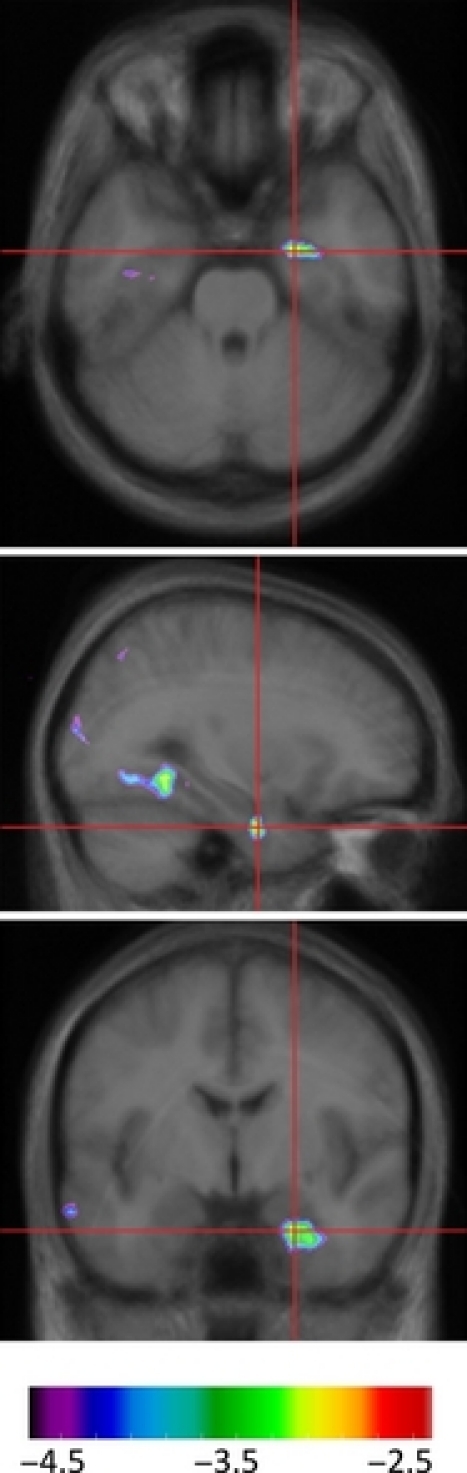
fMRI activity during the early encoding phase (experimental trial 1). Statistical parametric maps showing engagement of the hippocampus in the Val group during early learning (experimental trial 1). The *t*-statistic maps are superimposed on the anatomical average of all participants and displayed in the axial, sagittal and coronal planes. Cross-hairs are centred on the voxel with the highest BOLD response activity in the hippocampus for the Val group (*x* = 23, *y* = −6, *z* = −27; *t* = 4.05). No other region of the brain crossed the threshold for significance corrected for multiple comparisons. The colour bars illustrate the range of *t*-statistical values shown. For interpretation of references to color in the figure legend, please refer to the Web version of this article.

During the first experimental trial of the encoding phase (Stage 1), when participants were first exposed to the pathways and asked to actively memorize which of each pair of pathways contained an object, activation of the hippocampus was observed in the Val group (*x* = 30, *y* = −9, *z* = −32; *t* = 4.166, *P* < 0.0005) but not in the Met group (maximum peak: *x* = 21, *y* = −17, *z* = −20; *t* = 1.904) (Fig. 5). During the first experimental trial, no significant caudate nucleus activations were observed for either the Val group or the Met group. Hippocampal activity early in learning is consistent with our previous results (Iaria *et al.*, 2003) and studies in rodents (Packard & McGaugh, 1996).

**Fig. 6:**
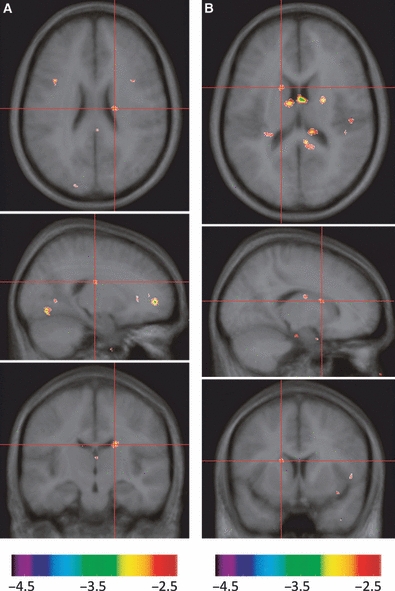
fMRI activity during the late encoding phase (the last two experimental trials of the encoding phase) of the Val > Met contrast. Statistical parametric maps showing (A) increased engagement of the caudate nucleus in the Met group as compared with the Val group during late learning (last two experimental trials before the recombined condition) and (B) increased engagement of the caudate nucleus in the Met group as compared with the Val group during the all-open test phase condition. The *t*-statistic maps are superimposed on the anatomical average of all participants and displayed in the axial, sagittal and coronal planes. Cross-hairs are centred on the voxel with the highest BOLD response activity in the caudate nucleus for the Met group (*x* = 22, *y* = −18, *z* = 26; *t* = 4.23). In the late learning contrast (A), the cross-hairs are centred on the voxel with the greatest degree of activation in the caudate nucleus (*x* = 20, *y* = −19, *z* = 24; *t* = −3.14). In the all-open contrast (B), the cross-hairs are centred on the voxel with the greatest degree of activation in the caudate nucleus (*x* = −6, *y* = 8, *z* = 16; *t* = −3.30). No other region of the brain crossed the threshold for significance corrected for multiple comparisons. The colour bars illustrate the range of *t*-statistical values shown. For interpretation of references to color in the figure legend, please refer to the Web version of this article.

By the end of the encoding phase (the last two experimental trials before moving to the test phase), when contrasting the Val group and the Met group directly, we observed negative activation in the caudate nucleus (*x* = 20, *y* = −19, *z* = 24; *t* = −3.14, *P* < 0.005; Fig. 6A), suggesting that the Met group activated this structure to a greater extent than did the Val group during late encoding.

Differential recruitment of memory systems between genotype groups was conserved during the all-open post-learning test phase (during the all-open condition, Stage 3). A group contrast of Val vs. Met revealed strong negative activation in the caudate nucleus, the same brain region that was found to differ between groups late in the encoding phase (*x* = −16, *y* = 8, *z* = 16; *t* = −3.3, *P* < 0.005; Fig. 6B). The data suggest that the Met group continued to preferentially recruit the caudate nucleus more than the Val group during the last test phase.

We observed robust fMRI activity in the parahippocampal cortex during both the learning and test phases of the CSDLT. Although these peaks are outside the regions defined *a priori* on the basis of our hypotheses, and therefore do not cross the Bonferroni threshold corrected for the entire brain, they are worth mentioning here because of the known anatomical projections of this structure to both the hippocampus and caudate nucleus. Maximum peaks were observed during learning (Val group maximum parahippocampal cortex peak, *x* = 28, *y* = −46, *z* = −9; *t* = 4.72; Met group maximum parahippocampal cortex peak, *x* = 15, *y* = −35, *z* = −4; *t* = 3.66) and during the all-open test condition (Val group maximum parahippocampal cortex peak, *x* = 38, *y* = −48, *z* = −5; *t* = 4.18; Met group maximum parahippocampal cortex peak, *x* = 18, *y* = −38, *z* = −2; *t* = 3.96). During the recombined condition of the test phase (Stage 2), no voxels in the hippocampus or caudate nucleus crossed our threshold for significance for either the Val group or the Met group.

Importantly, no performance differences were observed on the CSDLT between genotype groups as measured by trials to criteria during the encoding phase (Stage 1), errors on the recombined pairs (Stage 2), or total number of errors made during the all-open condition (Stage 3). Furthermore, no differences were found in the proportions of spatial and response learners between the genotype groups. This demonstrates that performance on the CSDLT and initial strategy cannot explain the differences observed in fMRI activity, but rather that these differences are related to genetic factors. Furthermore, it shows that individuals with lower fMRI activity in the hippocampus owing to the presence of a Met allele can compensate behaviourally for this by making use of the alternative response strategy.

Although there were no differences in CSDLT performance between genotype groups, performance on the CSDLT, recombined condition of the test phase (Stage 2) did differ between those who spontaneously used spatial and response strategies on the 4/8VM. An independent samples *t*-test showed that, when pairs were recombined during Stage 2, those who had used a spatial strategy on the 4/8VM had a higher percentage of correct pathway choices (81.25% correct; SEM, 7.1) than those who had used a response strategy (46.10% correct; SEM, 7.74) (*t* = 3.092, *P* < 0.006). These results demonstrate a significant association between strategies on the 4/8VM and on the CSDLT. In other words, our phenotype measure of spatial or response learning strategy is highly reliable across tasks.

## Discussion

Evidence from the behavioural and fMRI experiments in this study suggests that the presence of the Met allele at codon 66 of the BDNF gene is associated with a greater probability of using a caudate nucleus-dependent response strategy, at the expense of a hippocampus-dependent spatial strategy, in our virtual navigation memory tasks. This biasing effect was seen in the breakdown of spontaneous strategies on the 4/8VM and in fMRI activation during the CSDLT, two tasks that can be performed with either a hippocampus-based spatial or a caudate-based response strategy. Furthermore, participants using a given strategy in the 4/8VM were more likely to use the same strategy in the CSDLT, showing high reliability of our phenotype across the two tasks. These findings support previous results showing that the Met allele is associated with decreased performance and fMRI activity during tasks that require the use of hippocampus-dependent episodic memory (Egan *et al.*, 2003; Hariri *et al.*, 2003; Dempster *et al.*, 2005; Hashimoto *et al.*, 2008).

Whereas the presence of a single Met allele leads to an increased likelihood of using a hippocampus-independent response strategy, the effect of two Met alleles on spontaneous strategies is even more striking (Fig. 4). This supports the findings of Hashimoto *et al.* (2008), who suggested that the effect of the Met allele on hippocampus-dependent memory is dose-dependent. Similarly, Egan *et al.* (2003) found that Met/Met homozygotes scored considerably worse on their measures of episodic memory than either their Val/Val or Val/Met counterparts. The pattern that emerges in our data suggests that although the Val/Met genotype is worse for hippocampal function than the Val/Val genotype, the presence of at least one Val allele rescues some function when compared to Met/Met individuals.

The availability of two potential parallel strategies in our tasks is unique relative to the experimental design of previous Val66Met studies (Egan *et al.*, 2003; Hariri *et al.*, 2003; Dempster *et al.*, 2005). The use of the 4/8VM and CSDLT allowed us to study the effects of the Val66Met polymorphism on the interaction between multiple memory systems. The spontaneous quantifiable variability in our phenotype increases the sensitivity to differences in genetic variability. Whereas the use of hippocampus-dependent episodic memory tasks has allowed others to demonstrate impairments in Met carriers, we have shown that the effect of the Met allele on the hippocampus makes a participant less likely to use a spatial strategy when another option is available to them. The fact that 4/8VM and CSDLT performance measures did not differ between genotype groups is critical to exclude differences in performance as a confounding factor in our fMRI study. It also suggests that participants can compensate with the response strategy when spatial memory is at a disadvantage. This is an important strength of our study; the availability of strategies based on different memory systems has greater ecological validity, because it models the strategies available in people’s everyday lives, and demonstrates compensation mechanisms used by Met carriers.

Patients with selective thermo-coagulation lesions to the hippocampus have previously been shown to be impaired on the RAVLT and Rey–Osterreith Complex Figure tasks (Bohbot *et al.*, 1998). In the present study, healthy young participants with the Met genotype did not show any impairment on these tasks, indicating that they are still able to use their hippocampus despite the decreased functionality of BDNF. Although these neuropsychological tasks are well suited for detecting lesion-dependent impairments in memory, they are not as sensitive as spatial memory tasks. Other studies have shown performance differences between homozygous Val individuals and Met carriers on specific episodic memory-related subtests such as the story recall and logical memory subtests of the Wechsler Memory Scale (Egan *et al.*, 2003; Dempster *et al.*, 2005) and episodic memory recognition for scenes (Hariri *et al.*, 2003).

The fact that an effect of genotype on spontaneous strategies was demonstrated when no effect was detected on standard neuropsychological tests indicates that the navigational strategies in our study are more sensitive to BDNF genotype differences than standard tests of episodic/declarative memory. The fact that differences were observed in spontaneous strategies but not in any measure of cognitive performance highlights the utility of analysing behavioural processes at a finer level of detail in order to elucidate the often subtle link between genes and behaviour.

Whereas earlier studies demonstrated a direct effect of 4/8VM spontaneous strategies on fMRI activity during task performance (Iaria *et al.*, 2003; Bohbot *et al.*, 2004), the present study shows that the Val66Met genotype also predicts fMRI activity in the same brain regions on a similar task. One question that cannot be answered on the basis of the current limited sample is whether the strategy used and genotype have independent effects on fMRI activity. Future studies should investigate whether these effects interact with one another, or whether they independently explain the observed differences in brain activity.

Previous studies have demonstrated that acute and chronic stress can bias strategies towards the caudate nucleus-dependent response strategy in humans (Schwabe *et al.*, 2007, 2008; Schwabe & Wolf, 2009) and rodents (Kim *et al.*, 2001), a process that depends on an intact amygdala (Kim *et al.*, 2001) and is mediated by stress-induced increases in cortisol levels (Schwabe *et al.*, 2010). An interaction with BDNF expression may represent one potential mechanism by which stress impacts on spontaneous navigational strategy. Chronically stressed rats show a marked decrease in BDNF mRNA expression in the dentate gyrus and hippocampus (Smith *et al.*, 1995) and concomitant impairment on the hippocampus-dependent Morris water maze task (Song *et al.*, 2006), just as genetically modified mice with reduced BDNF expression display poor water maze performance (Heldt *et al.*, 2007). Direct infusion of BDNF into the hippocampus before and during chronic stress rescued water maze performance in rats who had been subjected to repeated immobilization stress (Radecki *et al.*, 2005).

It is possible that stress plays an intermediate modulatory role between genotype and behaviour, whereby the impact of BDNF on learning and memory strategies may be influenced by exposure to stress. Having the Val/Val genotype may increase the likelihood of participants using a spatial strategy, even following stress exposure, via increased BDNF expression and long-term potentiation in the hippocampus. On the other hand, participants with the Met allele would be more likely to use response strategies in their everyday lives, following stress exposure. Increased BDNF expression in Val/Val individuals could potentially protect against the demonstrated neurotoxic effects of cortisol on the hippocampus following stress (McEwen, 1998). This hypothesis is supported by the fact that the Val66Met genotype does not perfectly predict spontaneous learning strategies, suggesting that other factors, such as stress or polymorphisms in other genes, may also play a role.

Whereas genetic factors, modulated by external factors such as stress, play a role in predisposing individuals towards the spontaneous use of one strategy over another, it is probably the reinforcing effect of continually activating one of these memory systems that leads to differences in function and morphology between individuals over time. Previous studies have demonstrated that spatial memory training in taxi drivers can lead to increased grey matter in the hippocampus (Maguire *et al.*, 2000, 2006). Further support for the impact of training on brain morphology comes from studies in adult mice, which have demonstrated that training on either a spatial-dependent or a response-dependent task leads to significant growth in the hippocampus and striatum, respectively (Lerch *et al.*, 2007). The fact that the hippocampus can grow as a result of spatial memory training is important in the light of evidence linking hippocampal atrophy to psychiatric and neurological brain pathologies, such as Alzheimer’s disease (Bremner *et al.*, 2000; Apostolova *et al.*, 2006). Further evidence regarding the impact of genetic and environmental factors on the function and grey matter of the hippocampus will help to determine the risk of neurological and psychiatric illnesses before symptoms occur.

Although the mechanisms by which the polymorphism confers differences in disease risk remain to be worked out, gene association studies have suggested that Met carriers are at increased risk for a number of neurological and psychiatric pathologies known to affect the hippocampus, including Parkinson’s disease (Momose *et al.*, 2002), bipolar disorder (Neves-Pereira *et al.*, 2002), eating disorders (Ribases *et al.*, 2003), and obsessive compulsive disorder (Hall *et al.*, 2003). A neuroprotective effect of BDNF against Alzheimer’s disease has also been demonstrated in rodents and non-human primates (Tapia-Arancibia *et al.*, 2008; Nagahara *et al.*, 2009). The knowledge that spatial memory training can reverse atrophy of the hippocampus, in combination with knowledge of genetic and environmental risk factors for these conditions, may provide us with the tools required to selectively overcome predispositions to certain types of disorder.

The present study demonstrates that the BDNF Val66Met polymorphism plays a role in spontaneous navigational strategy. Met carriers demonstrated a decreased probability of using a hippocampus-dependent spatial strategy as compared with homozygous Val individuals, a phenomenon that could have long-term effects on grey matter and function in this brain structure (Bohbot *et al.*, 2004, 2007). These strategy differences translated into differences in brain activation between the Val and Met groups, with Val individuals preferentially activating their hippocampus and Met carriers exhibiting enhanced caudate nucleus activation with increased use of response strategies over time. Although genetic factors alone do not explain the differences in behaviour between spatial and response learners, the present study demonstrates that the BDNF gene with the Val66Met polymorphism is a novel candidate gene involved in modulating spontaneous strategies during navigation behaviour.

## Abbreviations

4/8VM: four-on-eight virtual maze task
BDNF: brain-derived neurotrophic factor
BOLD: blood oxygen level-dependent
CSDLT: concurrent spatial discrimination learning task
fMRI: functional magnetic resonance imaging
RAVLT: Rey Auditory Verbal Learning Task
SEM: standard error of the mean
